# Atypical and incomplete Kawasaki disease

**DOI:** 10.1186/1824-7288-41-S2-A45

**Published:** 2015-09-30

**Authors:** Maria Cristina Maggio, Giovanni Corsello

**Affiliations:** 1 University Department Pro.Sa.M.I. “G. D'Alessandro”, University of Palermo, Palermo, 90134, Italy

## 

Incomplete Kawasaki disease (KD) occurs in patients with fever lasting five or more days and with two or three of the classical findings (exanthema, conjunctivitis, changes in the extremities, erythema of oral mucosa and lips, cervical lymphadenopathy) [1]. It is difficult to define its real incidence.

However the association between bilateral conjunctival bulbar injection and perineal erythema, with early desquamation in a patient with platelet count > 450.000 after 7 days of fever is indicative of incomplete KD.

The definition “atypical KD” should be reserved for patients who have clinical manifestations such as renal impairment, unilateral peripheral facial nerve palsy, testicular swelling, pulmonary nodules and/or infiltrates, pleural effusions, diarrhea, vomiting and abdominal pain, acute surgical abdomen, hemophagocytic syndrome that generally are not seen in Kawasaki disease [1].

The “classical” clinical criteria have low sensitivity and specificity and therefore, other clinical and laboratory features may be helpful in establishing the diagnosis, especially for atypical or incomplete KD.

In the last years many studies highlighted clinical and/or laboratory findings useful to obtain a precocious diagnosis in atypical and incomplete KD, because coronary artery lesions (CAL) occur in 15-25% of untreated KD and approximately in 5% of KD who received intravenous immunoglobulin (IVIG) treatment before 10 days of fever [2,3].

Relief of hyponatremia, hypoalbuminemia, increased D-Dimer are biochemical findings useful for diagnosis [4].

The association of more parameters, also considering AST, ALT, gamma-GT, leukocytes, percentage of neutrophils, platelet counts, CRP, ESR can be a help in the labyrinth of KD. In fact a precocious diagnosis and a timely treatment with IVIG significantly improve prognosis and reduce the risk of CAL.

However the doubt of an atypical KD requests electrocardiography and echocardiography, to exclude CAL, pericardial effusion, valvular insufficiency, arrhythmia, prolonged PR interval, nonspecific ST and T wave changes in patients with FUO. Although aneurysms are rare before day 10 of fever, perivascular brightness, coronary arteries ectasia in the acute stage, indicate coronary arteritis before the aneurysms develop.

incomplete KD should be considered in all children with unexplained fever for ≥5 days associated with 3 or less of the clinical criteria. incomplete KD is more common in young infants, who have a higher risk to develop CAL. Young infants may present with incomplete KD: echocardiography should be considered in infants younger than 6 months with fever for ≥7 days, laboratory evidence of systemic inflammation, no other explanation of the disease.
